# 

**Published:** 1857-03

**Authors:** 


*Mich. State Med. Society.— Correction.*—The next annual meeting of this society will be held at the College Building, in Ann Arbor, March 25th, at 10, A. M., instead of the 24th, as previously stated, notice having been received that the Commencement will take place on the 26th instead of the 25th, as was supposed.

E. P. Christian, Secretary.

